# Imaging in Gastric Cancer: Current Practice and Future Perspectives

**DOI:** 10.3390/diagnostics13071276

**Published:** 2023-03-28

**Authors:** Teresa Giandola, Cesare Maino, Giuseppe Marrapodi, Michele Ratti, Maria Ragusi, Vittorio Bigiogera, Cammillo Talei Franzesi, Rocco Corso, Davide Ippolito

**Affiliations:** 1 Department of Diagnostic Radiology, IRCCS San Gerardo dei Tintori, Via Pergolesi 33, 20900 Monza, Italy; 2 Clinica Zucchi, Via Bartolomeo Zucchi, 20900 Monza, Italy; 3 School of Medicine, University of Milano Bicocca, Via Cadore 33, 20090 Monza, Italy

**Keywords:** stomach neoplasms, positron emission tomography computed tomography, tomography, X-ray computed, multiparametric magnetic resonance imaging, endoscopic ultrasound-guided fine needle aspiration, endoscopic ultrasound

## Abstract

Gastric cancer represents one of the most common oncological causes of death worldwide. In order to treat patients in the best possible way, the staging of gastric cancer should be accurate. In this regard, endoscopy ultrasound (EUS) has been considered the reference standard for tumor (T) and nodal (N) statuses in recent decades. However, thanks to technological improvements, computed tomography (CT) has gained an important role, not only in the assessment of distant metastases (M status) but also in T and N staging. In addition, magnetic resonance imaging (MRI) can contribute to the detection and staging of primary gastric tumors thanks to its excellent soft tissue contrast and multiple imaging sequences without radiation-related risks. In addition, MRI can help with the detection of liver metastases, especially small lesions. Finally, positron emission tomography (PET) is still considered a useful diagnostic tool for the staging of gastric cancer patients, with a focus on nodal metastases and peritoneal carcinomatosis. In addition, it may play a role in the treatment of gastric cancer in the coming years thanks to the introduction of new labeling peptides. This review aims to summarize the most common advantages and pitfalls of EUS, CT, MRI and PET in the TNM staging of gastric cancer patients.

## 1. Introduction

In recent decades, diagnostic imaging, particularly cross-sectional techniques including contrast-enhanced computed tomography (CT), magnetic resonance imaging (MRI), and positron emission tomography (PET), has assumed a central role in the diagnosis of various pathologic entities. Although all of these techniques should be considered interchangeable, each has advantages and pitfalls, while all have a more than predictable potential.

In current clinical practice, one of the most common goals is to stage cancer patients in order to best assess their treatment needs and guide them toward surgical or medical interventions [1].

In this context, all of the above techniques can be considered efficient tools for the staging of gastric cancer patients. However, not all of these imaging techniques can be considered interchangeable, and the choice should be made carefully based on international guidelines and the experience of one’s own center [1]. Due to continuous technical improvements, radiology plays a key role in determining all staging parameters, especially the tumor extent, nodal status, and the presence of distant metastases. Although cross-sectional imaging was considered useful for determining the nodal status and the presence of distant metastases in past decades, new technological improvements have greatly facilitated the preoperative staging of the tumor extent.

There are more treatment options available for GC patients, and therapeutic strategies depend on the tumor stage. For very early, superficial tumors (T1a), endoscopic mucosal resection/submucosal dissection is the preferred procedure, whereas for early-stage cancers that are not amenable to endoscopic resection, surgical resection is the treatment of choice. Total/distal gastrectomy, depending on the tumor location, in conjunction with neoadjuvant chemotherapy, is the standard treatment for locally advanced GC (≥T3, any N or ≥T2, N+). For advanced unresectable/metastatic GC (35–40% of cases at the time of initial diagnosis), chemotherapy is still considered the standard treatment [2]. Therefore, it is crucial to correctly determine the stage of disease in order to select the most effective therapeutic pathway, and imaging plays a pivotal role in this regard.

On this basis, the present review aims to summarize and report the main advantages and pitfalls of imaging techniques for the staging of gastric cancer patients, collect the main data reported in the current literature, highlight the main shortcomings in research and provide future perspectives.

## 2. Epidemiology, Risk Factors and Pathological Classification Systems

GC is the fifth most common type of cancer and the third leading cause of death worldwide. As previously reported [2], it is important to emphasize that gastric cancer is particularly common in East Asia, Eastern Europe and South America and is especially prevalent in men.

Previously, chronic infection with Helicobacter pylori was considered one of the most common pathological factors associated with gastric cancer. Nowadays, however, several pathological factors are considered to contribute to the development of GC, including age, cigarette smoking, alcohol consumption and pernicious anemia. In addition, the consumption of salted foods has been shown to be a risk factor for H. pylori infection [3]. Finally, approximately 10% of all gastric cancer patients have a familial clustering due to germline mutations [4].

Since 1971, early gastric carcinoma (EGC), defined as a tumor that does not invade deeper than the submucosa and is independent of nodal metastasis (T1, any N), has been pathologically classified into three different macroscopic manifestations, including the protrusive (type I), superficial (type II) and excavated (type III) types. In addition, type II is divided into raised, shallow and depressed types [5].

From a macroscopic perspective, gastric cancer staging is routinely performed using the TNM staging system, 8th Edition of the AJCC [6].

The Japanese Gastric Cancer Association classification should be considered when classifying local-regional nodules, which are divided into perigastric and extraperigastric types. Nodal status is one of the most widely accepted prognostic factors related to overall survival: reported five-year survival is directly proportional to the N stage (86.1% for N0 and 5.9% for N3) [7].

GC can spread via the lymphatic system to the perigastric ligaments, mesentery, omentum and adjacent and distant organs [8] and via vascular structures and nerves.

The presence of distant metastases is a contraindication to surgical resection, and detection is paramount to the guidance of treatment. Secondary liver involvement is the most common site of spread, followed by the lungs, bones and adrenal glands. The involvement of retro-pancreatic, mesenteric root, mid-colonic, para-aortic, peripancreatic, infra-diaphragmatic, para-esophageal, lower thoracic and other distant nodes is considered to represent metastatic (M1) disease. Sites of distant metastases include supraclavicular (Virchow node), periumbilicular (Sister Mary Joseph node) or the left axillary node (Irish node).

## 3. Diagnostic Techniques

To date, the preferred imaging modalities for the staging of GC before surgery are CT and endoscopic sonography (EUS). EUS has been used as the tool of choice for locoregional staging because of its ability to differentiate between the layers of the gastric wall and its high accuracy in terms of distinguishing EGS from deeper lesions [9,10]. On the other hand, several reports have pointed out one of the most important pitfalls, which is the underestimation or overestimation of invasion, which is influenced by inflammation around the lesion. The second most important pitfall concerns the evaluation of distant nodules, which is difficult due to the limited depth of invasion, while distant organ metastases cannot be evaluated [9,11].

Conversely, CT was initially used to detect distant metastases in recent decades [12], and over time, it has also played an increasingly important role in evaluating the extent of locoregional disease [13,14,15]. Even though it is characterized by a higher spatial resolution, CT also has a low diagnostic value [16].

### 3.1. Endoscopic Ultrasonography (EUS)

EUS is a combined technique used for endoscopy and high-frequency ultrasound (5–12 Hz) that provides high-resolution images with a limited penetration depth (between 1 and 6 cm). Dilatation of the lumen (200–400 mL) with water may contribute to a better assessment of the gastric walls.

The normal gastric wall is presented as a 5- to 9-layered structure [17], according to the high resolution of the probes: not all layers correspond to the histological ones, since some of them can present echoes due to interfaces. The two inner layers (hyper and hypo-echoic, respectively) represent the superficial mucosa and the muscularis mucosa. The 3rd (hyperechoic) layer corresponds to the submucosa, the 4th (hypoechoic) to the muscularis propria, and the 5th (hyperechoic) to the serosa, which is usually not easily distinguishable from the surrounding hyperechoic adipose tissue.

Nowadays, there is no consensus on the normal thickness of the gastric wall, but 2–4 mm should be considered the normal range [18].

GC usually presents as inhomogeneous hypoechoic wall thickening that is focal or diffuse, affects one or more layers, has possible growth outside the wall and eventually infiltrates other structures [17].

#### T and N Staging

The overall accuracy of EUS for T staging ranges from 65 to 92.1%. In particular, the sensitivity and specificity for serosa involvement range from 77.8 to 100% and from 67.9% to 100%, respectively [9]. By grouping GC according to the WHO classification, the sensitivity for more invasive tumors increases and ranges from 88.1% for T1 to 99.2% for T4 [19].

Although EUS is considered the imaging modality of choice for locoregional staging of GC, it has several limitations. First, it is an operator-dependent technique that is invasive and is associated with sedation-related complications. In addition, not all gastric regions can be easily assessed, and special attention is paid to the lesser curvature, subcardiac region and gastroesophageal junction. The same problems occur with extensive ulceration and with large lesions [20].

Nodal metastases are visualized on EUS as roundish, hyperechoic metastases located in perigastric zones. The overall accuracy of EUS in N staging generally ranges from 66 to 90% [21,22] with low sensitivity values for stages N2 and N3 [21]. One of the most important advantages of EUS in N staging is the possibility of fine needle aspiration (EUS-FNA), which contributes to the improvement of the overall accuracy. In this regard, the sensitivity, specificity and positive predictive value of EUS-FNA increase to 92%, 98% and 97%, respectively [23].

In addition, EUS has a limited depth of penetration and is therefore of limited use in the evaluation of distant metastases, which are usually investigated by other diagnostic methods [23].

Table 1 summarizes the most important studies regarding the usefulness of EUS in the staging of GC patients.

### 3.2. Computed Tomography (CT)

Before performing CT, the patient must be fasting for at least 6 h, and pharmacological hypotonization is achieved with 10–20 mg of butylscopolamine bromide administered intramuscularly or intravenously 10 to 15 min before the examination [24].

To achieve optimal gastric distension, negative (air) or neutral (water or methylcellulose) contrast agents are usually used to better visualize the enlargement of each layer of the gastric wall [25].

The administration of intravenous contrast medium is mandatory for the examination of the gastric walls. CT images should be acquired at least in the unenhanced phase and approximately 70 s after injection, the optimal time for GC enhancement. To assess the presence of vascular variants of the stomach, arterial phase imaging can be added [26]. Finally, postprocessed reconstructions (multiplanar reconstructions—MPR) in the coronal and sagittal planes can provide a better assessment of the tumor location and depth.

Virtual gastroscopy (VG) is a CT-reconstructed three-dimensional (3D) endoluminal image set that simulates an endoscopic view. For VG, air is the preferred oral contrast agent. Limitations of this technique include the additional time (10 to 20 min) required to process the images and the higher level of technical expertise needed.

Normal gastric walls show a multilayered pattern with an inner enhancing layer that histologically corresponds to the gastric mucosa. The intervening hypoattenuating layer represents the submucosa, and the outer, slightly hyperattenuating layer of variable thickness corresponds to the muscularis propria and serosa layer [27].

Gastric cancer presents as focal or diffuse wall thickening characterized by inhomogeneous enlargement that destroys normal gastric wall structures [27]. Therefore, the size of the gastric wall thickening and the degree of enhancement may affect the detection rate and accuracy of T-staging. In particular, focal thickening of greater than 5 mm in a well-expanded stomach is considered a neoplastic lesion [28].

#### 3.2.1. T and N Staging

**Figure 1:**
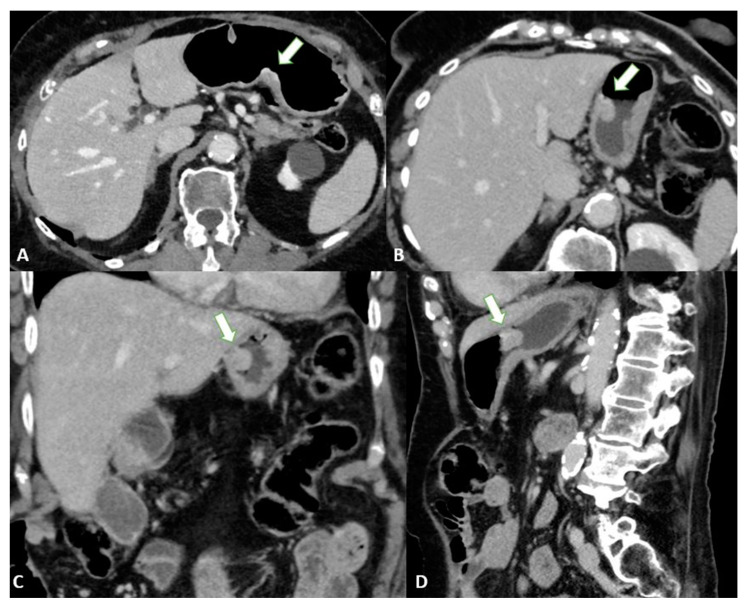
T2 gastric cancer in a 67-year-old-female patient. (**A**) Axial 2D image in the portal venous phase showing enhanced wall thickening in the lesser curvature side of the low body of the stomach (white arrow). T2 gastric cancer in a 66-year-old-male patient. (**B**) Axial 2D image (**C**) Coronal 2D image and (**D**) Sagittal 2D image showing enhanced wall thickening (white arrow) in the lesser curvature side of the middle body of the stomach. In both the patients, the tumor invades the muscularis propria layer.

T1a tumors are usually not visible, whereas T1b tumors show mucosal thickening and enhancement. The distinction between T1b and T2 can be made based on the appearance of the thickening base: T1b shows a faint attenuated stripe indicating the submucosal layer, whereas T2 shows a loss of this layer due to the involvement of the submucosa [24] (Figure 1).

**Figure 2:**
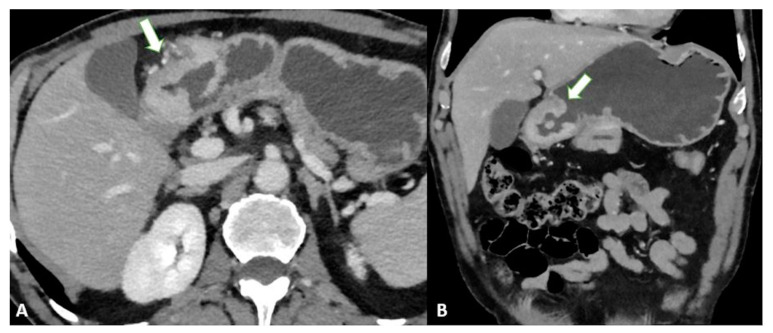
T3 gastric cancer in a 72-year-old male patient. (**A**) Axial 2D image in the portal venous phase and (**B**) Coronal 2D reconstruction showing wall thickening (white arrow) in the lesser curvature of the low body of the stomach and inhomogeneous enhancement. The tumor invades the subserosa layer without invasion of the serosa and adjacent structures.

**Figure 3:**
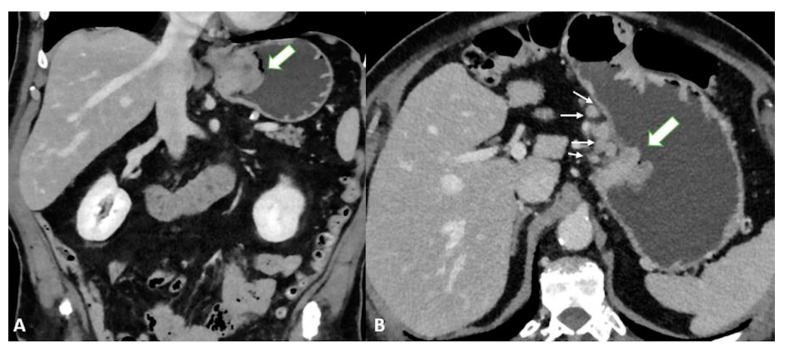
T3 gastric cancer in a 74-year-old female patient. (**A**) Coronal 2D reconstruction in the portal venous phase and (**B**) Axial 2D image showing (thick arrows) enhanced wall thickening in the lesser curvature side of the high body of the stomach. (**B**) also shows a cluster of pathologic round lymph nodes adjacent to the gastric cancer (thin arrows). The tumor invades the subserosa layer without invasion of the serosa and adjacent structures.

T3 tumors have a subserosal invasion, and discrimination between a gastric mass and the outer layer can be difficult: small linear strandings in the gastric fat, due to a desmoplastic reaction, can be confused with serosal involvement (Figure 2 and Figure 3). 

**Figure 4:**
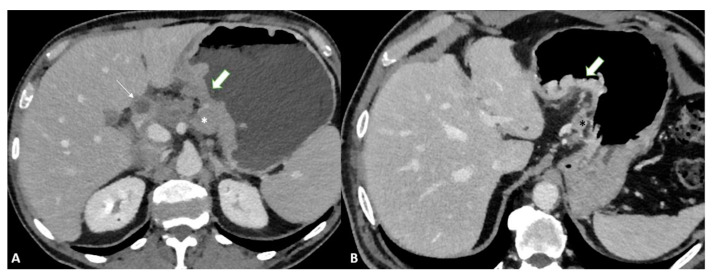
(**A**) Axial 2D image in the portal venous phase of a T4a gastric cancer in a 66-year-old female patient. The tumor (thick arrow) is the enhanced wall thickening in the lesser curvature side of the middle body of the stomach which penetrates the serosa with some solid deposits (white asterisk) in the perivisceral fat tissue and some pathologic lymph nodes (thin arrow) with necrotic-colliquative components inside. (**B**) Axial 2D image of a T4a gastric cancer in a 78-year-old female patient. The tumor (thick arrow) is the enhanced wall thickening on the lesser curvature side of the low body of the stomach which penetrates the serosa with some spiculatures in the perivisceral fat tissue and a pathologic lymph node (black asterisk).

Finally, T4a also demonstrates serosal involvement, which makes the differential with T3 very difficult. This is especially true because the gastric serosa is not well defined due to the different amounts of subserosal adipose tissue. To solve the differential, T4a frequently shows solid nodules or band-like stranding in the perivisceral adipose tissue (Figure 4). 

**Figure 5:**
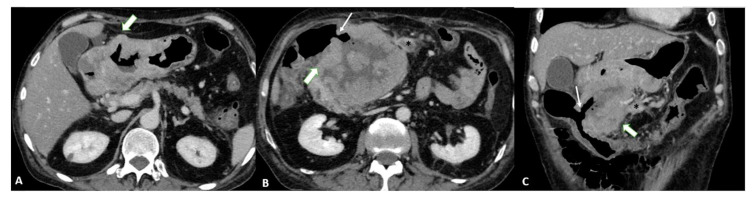
T4b gastric cancer in a 74-year-old female patient. (**A**,**B**). Axial 2D images in the portal venous phase and (**C**) Coronal multiplanar reconstruction showing (thick arrows) a bulky tumor of the middle-low body of the stomach and of the gastric antrum with necrotic-colliquative components inside, ulcerative alterations and some solid deposits (black asterisks) in the perivisceral fat tissue. The tumor fistulizes and infiltrates the transverse colon (thin arrows).

T4b shows extension into an adjacent structure and shows the loss of the fat plane between the gastric mass and adjacent organs (Figure 5).

Table 2 summarizes the most common CT features for each T category.

Based on the abovementioned multilayered appearance of the gastric wall, several studies have suggested CT as a useful tool for T staging [22,23,27,28,29]. An analysis of the main studies published in the literature showed that the overall diagnostic accuracy for T staging ranges from 77 to 89%. Considering its importance for management, the sensitivity and specificity of serosal invasion have been evaluated in detail with values ranging from 82.8 to 100% and from 80 to 96.8%, respectively [23].

**Figure 6:**
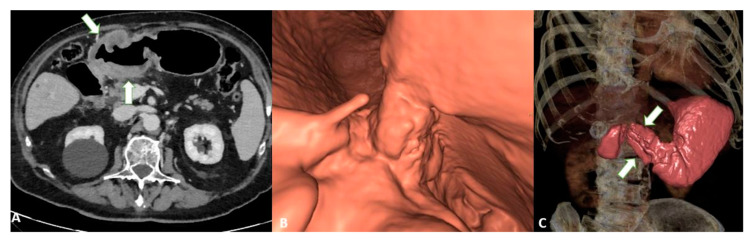
T4a gastric cancer in a 53-year-old female patient. (**A**) Axial 2D image in the portal venous phase with distension of the gastric lumen with air shows a bulky circumferential tumor (white arrows) of the low body of the stomach and of the gastric antrum with ulcerations; (**B**) Virtual gastroscopy delineates a lesion protruding in the lumen of the stomach; (**C**) Computed tomography gastrography shows a mucosal irregularity (white arrows) with a reduction of the lumen of the stomach.

The added value of MPR combined with VG can increase the overall accuracy (from 73 to 89%) thanks to its ability to better assess invasion (Figure 6) [30].

**Figure 7:**
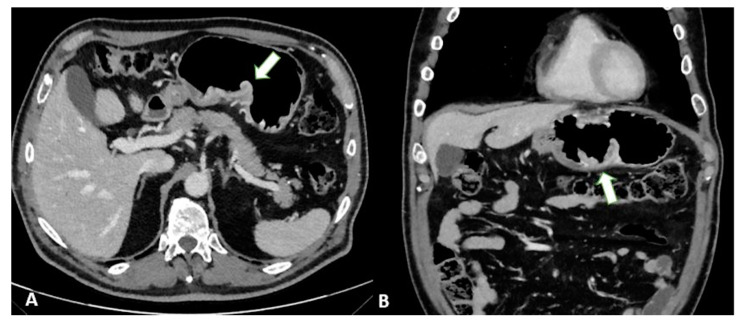
T3 gastric cancer in a 68-year-old female patient. Contrast enhanced CT with gastric distension using air. (**A**) Axial 2D image and (**B**) Coronal 2D image in the portal venous phase with distension of the gastric lumen with air showing a semicircumferential tumor (white arrows) of the low body of the stomach with ulcerations.

To confirm this hypothesis, other studies have reported that VG can enhance the recognition of GC, especially EGC [31]. In support of these data, it has been demonstrated [32] that VG can increase the overall performance compared to axial images alone in detecting EGC with sensitivity and specificity levels of 91.9% and 74% and 62.9% and 82.9%, respectively (Figure 7).

Similarly, in a study involving 106 patients, GC was found to be easier to detect with VG (87% vs. 98%). The authors demonstrated that the accuracy was significantly higher when using VG compared with axial images (84% and 77%, respectively). On the other hand, the authors reported no significant difference in N staging, with overall accuracy levels of 62% and 64% for axial images compared to VG, respectively [24].

The differential diagnosis between stages T2 and T3 or T3 and T4 by CT has long been controversial. In addition, using CT to determine gastric cancer at the T3 stage is more difficult when the imaging of the serosa of the intestine shows irregular, protruding bands. In this case, MRI could be a reliable diagnostic tool to help with radiology assessments, especially for distinguishing T2 from T3 [33].

**Figure 8:**
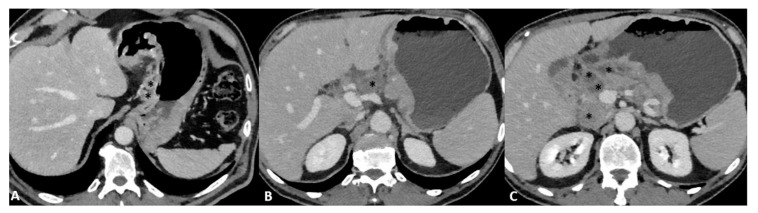
Axial 2D images in the portal venous phase show different pathological node locations (black asterisks): (**A**) pathological lymph nodes in the lesser curvature of the stomach; (**B**) pathological lymph nodes of the lesser curvature and the celiac artery; (**C**) pathological lymph nodes of the pancreatic head and para-aortic region. The lymph nodes in the images present an inhomogeneous contrast enhancement with central hypodensity due to the presence of necrotic components inside.

To distinguish normal from pathologic nodes, size is the most accepted parameter. However, there is no clear consensus on the threshold size for suspicious nodes. The threshold size depends on the location and ranges from 6 to 10 mm in the upper abdomen [34]. Other characteristics of metastatic nodes include a round shape, a cluster of more than three nodules, and the degree and heterogeneity of enhancement [35] (Figure 8).

In addition, microscopic metastases can often be found in normal-sized nodes in patients with EGC, which reduces the accuracy of N staging in EGC compared with advanced cases [24].

According to a meta-analysis [10], the CT sensitivity and specificity of N staging range from 62.5 to 91.9% and 50.0 to 87.9%, respectively. These diagnostic values are associated with an acceptable accuracy level (86.3%), but the authors [36] highlighted an important problem with N-staging based on the mediocre to good inter-reader reliability (κ 0.449–0.662) in classifying the nodal status.

Nodal staging with MPR showed no significant improvement [23], with overall accuracy levels of 62% and 64% for axial images and MPR, respectively.

#### 3.2.2. M Staging

CT is considered the most important imaging technique for the detection of distant metastases and is still considered the preoperative reference standard, even in GC patients. In this regard, an overall M staging accuracy of 96.6% was reported in a study involving 350 patients [31]. In addition, the authors reported good sensitivity and specificity levels for the most common metastatic sites, including the peritoneum (90% and 97%), liver (80% and 99%), and pelvis (100% and 99%).

In particular, peritoneal carcinomatosis is one of the pathological entities that need to be evaluated for accurate M staging: One study reported sensitivity and specificity levels for detecting peritoneal carcinomatosis of 50.9% and 96.2%, respectively [37]. In another study [38], the authors noted that regional nodal metastases, advanced gastric cancer, undifferentiated pathology and the presence of ascites may be considered independent predictors of peritoneal carcinomatosis.

CT is still considered the tool of choice for peritoneal imaging and is included in the ESMO guidelines for GC. However, it has limited soft tissue contrast, which limits its ability to visualize small peritoneal metastases (PM), especially when adjacent to bowel structures or the mesentery, limiting its sensitivity in detecting lesions and its accuracy in staging PM, as it underestimates the results compared with the surgical Peritoneal Cancer Index (PCI). For this reason, laparoscopy remains the reference for PC staging [39].

The sensitivity of CT for peritoneal carcinomatosis is low to moderate and varies widely from 23 to 76%. Sensitivity is particularly low for small lesions (from 11% in <5 mm lesions compared with 94% for >5 cm lesions) and at specific sites, such as the mesentery, diaphragmatic borders, and bowel walls [40].

Table 3 summarizes the most important studies regarding the usefulness of CT for the staging of GC patients.

### 3.3. Magnetic Resonance Imaging (MRI)

In the past, MRI had a limited role in the evaluation of GC, especially because of the presence of motion artifacts, the long examination time, and the high cost [41].

However, in recent decades, major advances have been made in MRI technology that have improved the diagnostic performance in many areas of medicine, including oncology. These improvements include rapid breath-hold imaging techniques, abdominal bandage placement, the administration of anti-inflammatory drugs, and the use of phased array coils. MRI has the great advantage of providing superior soft tissue contrast and multiple imaging sequences without radiation-related risks. In addition, the high quality soft tissue imaging achieved with MRI allows the visualization of the anatomic wall layers [42].

However, the guidelines for the treatment of GC do not specify MRI as a possible imaging modality for staging. In addition, the most recent TNM guidelines do not recommend the use of MRI for the imaging assessment of T, N or M parameters in GC [43].

Although recommendations are not yet available, the use of butylscopolamine bromide for hypotension and the use of water as an oral contrast agent may be considered useful, as for CT. The fat-suppressed T1-weighted gradient echo sequence, T2 weighted images with single-shot fast spin echo or turbo spin echo, and true fast imaging with steady-state precession (true-FISP) are common sequences for the detection of gastric cancer [42].

#### TNM Staging

As mentioned above, CT can be considered a useful tool for the staging of GC patients. However, MRI should also be considered for these purposes. In fact, a meta-analysis [44] comparing the diagnostic value of the most common imaging modalities for the staging of GC was published in 2012. This showed that the overall accuracy in the T-stage assessment of MRI was statistically better than that of CT (82.9% ± 3.7% vs. 71.5% ± 2.7%). MRI also appeared to be better than CT in terms of sensitivity in assessing the N parameter of GC (85.3% and 77.2%, respectively).

Another meta-analysis [45] showed that the pooled sensitivity of MRI in diagnosing GC stages T1, T2, T3 and T4 was 66%, 85%, 86% and 88%, respectively, and it was 86% for correctly assessing the N parameter.

When analyzing the diagnostic values according to the T stage, some authors [46] reported that CT and MRI had accuracy levels of 37.5% and 50% for the T1 stage and 81.2% and 88.7% for the T2 stage, respectively; moreover, they showed no significant differences in accuracy in the evaluation of T3 and T4 lesions, suggesting that MRI may be more suitable for identifying EGC.

These aspects were confirmed in a similar study [47], which reported that MRI was superior for detecting T1 lesions compared with CT (50% vs. 37.5% accuracy for MRI and CT, respectively) [37], with overall accuracy levels of 60% and 48% for the T stage and 68% and 72% for the N stage, respectively.

Finally, a 2017 systematic review [48] found that both the specificity and sensitivity of MRI were greater than those of CT (86% vs. 83% and 88% vs. 86%, respectively), although without statistical significance.

**Figure 9:**
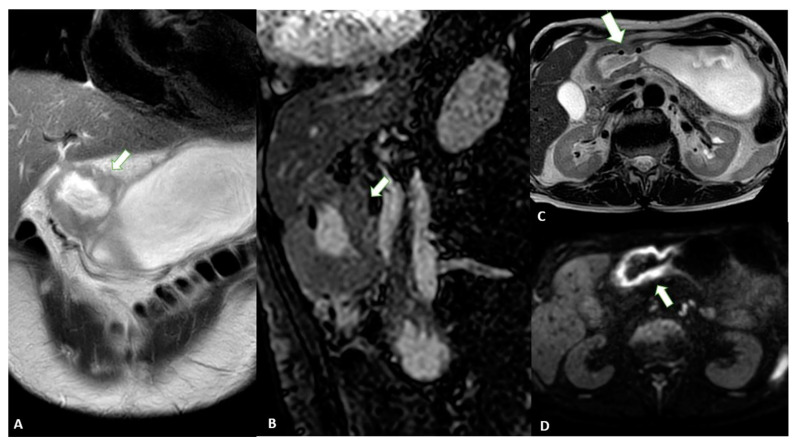
MRI images of a T3 gastric cancer of the gastric antrum in a 79-year-old male patient. (**A**) Coronal 2D image and (**C**) Axial 2D image of the Turbo Spin Echo (TSE) T2 sequence showing a circumferential lesion (arrow) invading the subserosa layer with an intermediate signal intensity; (**B**) Coronal 2D Balance Fast Field Echo (BFFE) sequence showing a circumferential lesion (arrow) with a low signal intensity; (**D**) Axial Diffusion Weighted Image (DWI) showing an area of signal restriction (arrow) corresponding to the tumor.

Advances in imaging techniques, such as the introduction of diffusion-weighted imaging (DWI), may provide important data for the definitive diagnosis of various pathologic entities. In this context, DWI can help to distinguish T4 from the lower stages of GC with a high reliability [49]. The authors reported a sensitivity of 92.1%, specificity of 75%, and accuracy of 89.1% for ≤T2 vs. ≥T3 lesions and a sensitivity of 75%, specificity of 88.5%, and accuracy of 82.6% for ≤T3 vs. T4 lesions in 46 patients (Figure 9).

Another study reported that the diagnostic accuracy of DWI in T staging, lymph node staging and distant metastasis is comparable to that of CT, with DWI performing better in the detection of nodal metastasis [50]. In addition, some authors [51] have highlighted the use of DWI in the assessment of the T stage in patients with gastric cancer. By evaluating 51 patients who underwent MRI, the authors demonstrated that DWI can significantly increase the overall detection accuracy for all lesions (88.2% vs. 76.5%, *p* = 0.031).

MRI is also an efficient tool for the diagnosis of distant metastases, such as those in the liver and peritoneum. A recent review and meta-analysis compared the diagnostic accuracy of MRI with hepatobiliary contrast with CT [52]: it was demonstrated that the sensitivity of MRI was significantly higher (sensitivity and specificity per lesion of 86.9–100.0% and 80.2–98.0% versus 51.8–84.6% and 77.2–98.0% for MRI and CT respectively). In addition, the authors demonstrated that the sensitivity of MRI increases for lesions smaller than 10 mm (RR = 2.21, 95% CI = 1.47–3.32, *p* < 0.001).

In addition, DWI should be considered a useful tool for the evaluation of peritoneal dissemination. Indeed, a recently published meta-analysis highlights the good diagnostic value for the detection of peritoneal carcinomatosis, with pooled sensitivity and specificity values of 89% (95% confidence interval [CI]: 83–93%) and 86% (95% CI: 79–91%), respectively. When included studies were grouped by primary tumor, a pooled sensitivity of 97% (95% CI: 68–100%) was reported for gastrointestinal malignancies [53].

Table 4 summarizes the most important studies regarding the usefulness of MRI in the staging of GC patients.

### 3.4. Comparison between Techniques

Recently, several studies have compared the two imaging modalities and demonstrated that CT has higher accuracy for T staging than EUS [29,54,55]. In this regard, since 2005 [54], it has been highlighted that the accuracy of CT in T-staging almost equals that of EUS and that CT could replace EUS for preoperative staging.

Similarly, in a study involving 227 patients [56], CT and EUS were shown to have similar T-staging accuracy levels in tumors without ulcerative portions, whereas CT performed significantly better for ulcerative tumors (*p* < 0.0001).

In agreement with the above studies, some authors [57] found that the accuracy of T staging with EUS and CT was 87.5% and 83.3%, respectively, whereas the accuracy of N staging was 79.1% and 75.0%.

Analogous results were reported [58] when comparing VG and EUS for the detection of gastric cancer. The authors showed that the prediction of the T stage was similar between the two techniques with accuracy levels of 82.2% and 83.7%, respectively. In another study in which MRI, CT, and EUS were performed in the same population of gastric cancer patients, the results showed the highest sensitivity for EUS (94%) compared with MDCT (65%) and MRI (76%), underscoring the primary role of this technique in detecting locally advanced tumors. Conversely, MRI and CT yielded significantly higher specificity levels, demonstrating that both techniques are better able to detect tumors without serous invasion [59].

In ulcerated EGC, the accuracy of EUS was shown to be lower compared to lesions without ulceration (30.8% vs. 93.3%), while CT showed no significant differences between them (61.5% vs. 86.7%) [60].

Finally, a diagnostic meta-analysis [61] was used to determine the accuracy of CT and EUS in the staging of GC. The results indicated that EUS is superior to CT for T1 staging (AUC 0.903 and 0.774, respectively), whereas no significant differences were found for T2–T4 lesions (AUC 0.845 and 0.793, 0.814 and 0.804, and 0.846 and 0.930 for T2, T3 and T4, respectively) or stage N1 (AUC 0.690 and 0.693, respectively). Subsequently, the sensitivity of CT was significantly higher for N2 (0.562 vs. 0.301) and N3 (0.211 vs. 0.162).

### 3.5. Positron Emission Tomography (PET)

Even through PET-CT is considered a useful diagnostic tool for different cancer types, there is no evidence or recommendations by the most important international guidelines that it is a necessary examination step in the staging of GC [62]. By searching the international literature, it is possible to understand the lack of experience worldwide, which may be due to the initial reports which stated that gastric tumors are frequently not fluorodeoxyglucose (FDG)-avid [62]. On the other hand, FDG-PET is useful, particularly for the detection of the node status and, consequently, to determine the best treatment option. In this setting, a recently published paper [63] demonstrated that the majority of patients have an FDG-avid tumor (80.6%) and that the T stage is strictly associated with the FDG-avidity (T2–3 OR = 3.38 while T4 OR = 7.46). On the other hand, the authors demonstrated that about 25% of nodes are FDG-avid. They finally concluded that the sensitivity and specificity for metastatic disease are more than acceptable (49.3% and 97.1%, respectively).

FDG-PET can play a role in determining the management of GC patients. More recently, some authors [64] conducted a systematic review of data from 11 studies representing more than 2000 patients from the last decade. The authors reported management changes in 3 to 29% of cases, while no studies reported the risk of recurrence or survival rates in patients staged with or without FDG-PET.

Even through FDG-PET has some limitations in the detection and T staging, different studies have investigated its importance in the evaluation of nodes. CT is known to be a reliable tool for identifying pathological nodes [35], even though it is not too robust, as previously mentioned. On the other hand, FDG-PET can be considered a reliable imaging examination method to identify small metabolically active nodes [65]. During the last year, a systemic review [65] aimed to determine the added value of FDG-PET in the detection of node metastases. The authors underlined that the SUVmax is a metabolic parameter that is commonly used to detect the node status. However, SUVmax is susceptible to the blood glucose concentration, the timing of the uptake, respiratory motion, and the interobserver variability. To endorse the usefulness of SUVmax, a published study that enrolled 151 patients with confirmed node metastasis demonstrated that 18% of the patients showed positive FDG uptake and, by using a cutoff value of 2.8, it was possible to predict relapse-free survival (RFS) and (OS) [66]. By combining pathological data and imaging features, they demonstrated that SUVmax can be considered an independent prognostic factor for OS (HR = 2.80).

Not only metabolic activity but also number counts can be used. In 2016, by retrospectively enrolling 50 patients, some authors [67] demonstrated that the number of metabolically positive nodes correlated with histological results (r = 0.694, *p* = 0.001). In the final model, the authors demonstrated that only surgical outcomes (R1 vs. R0) and the number of metabolically positive nodes (≤2 vs. ≥3) were independent factors for poor OS.

To move forward and better depict primary tumors, in recent years, the use of labeling peptides has been proposed with the introduction of 1,4,7,10-tetraazacyclodecane-1,4,7,10-tetraacetic acid (DOTA), a universal chelator that is capable of forming stable complexes with radiotracers of the metal Gallium (Ga). Moreover, different labeling peptides were developed to be taken up by gastric cancer cells with a special focus on fibroblast-activated protein (FAP) conjugated with DOTA. In this setting, different studies have reported that Ga-FAPI is taken up more intensely by tumors than FDG, resulting in a higher sensitivity for the detection of primary lesions and metastases [68]. In 2022, a study [69] enrolled 61 patients and compared FDG and Ga-FAPI. The authors demonstrated a higher positive detection rate for Ga-FAPI in comparison with FDG in the evaluation of primary tumors. On the other hand, they concluded that both modalities underestimated N staging compared with pathological N staging. Similar results were found in two studies that enrolled 35 and 25 patients with gastric cancer, respectively [70]. Moreover, one of them [70], demonstrated that Ga-FAPI exhibited a higher sensitivity level compared to FDG for the N status (97.4% vs. 42%) and the detection of distant metastases (97.2% vs. 43.1%). Similar results were reported regarding the node status, indicating that Ga-FAPI detected more positive nodes than FDG (637 vs. 407), even if both modalities underestimated them in comparison with pathological staging [70].

Finally, even though the application of PET is not yet recommended by international guidelines, all reported studies demonstrated its potential for detecting not only the primary tumor, reducing the false negative rate, but also nodal involvement and distant metastases.

## 4. New Frontiers

### 4.1. CT Volumetry

Recently, CT volumetric rendering techniques have been used to generate three-dimensional (3D) volume-rendering images, aiding in the calculation of the exact volumes of solid organs or tumors.

There are two CT volumetry methods. Two-dimensional volumetry is based on manually segmented regions of interest (ROIs): the final gastric volume is obtained by multiplying the ROI areas with the slice thickness. On the other hand, 3D volumetry is a semi-automatic segmentation process that requires dedicated post-processing softwares [71].

Based on these approaches, it has been demonstrated that CT volumetry results are comparable to EUS in terms of their accuracy for T2 to T4 staging (83% to 95%), and the tumor volume was proposed as adjunct information for the prediction of metastatic disease and the risk of peritoneal spread [72].

In another study, it was reported that the tumor volume of the oesophagogastric junction is an independent risk factor for node metastasis [73]. Based on these factors, some authors have demonstrated that the tumor volume, using pathological data as the reference standard, is correlated with survival, and it has been suggested as a significant prognostic factor [74].

### 4.2. Perfusion CT (pCT)

Perfusion Computed Tomography (pCT) is a minimally invasive technique that allows quantitative and qualitative evaluation of tissue perfusion by injecting iodinated contrast agents and performing consecutive acquisitions to estimate time enhancement curves. Some studies have proposed that pCT could be used for GC patients, and computed parameters can provide important information regarding tumor angiogenesis [75,76].

Indeed, it has been reported [75] that the blood volume of GC is significantly correlated with the microvessel density, which may be considered valuable information for preoperative assessment. Malignant tumors, including GC, might have leaky vessels produced by neoangiogenesis, and leaky vessels within the tumor can increase the permeability surface, an important data point that is deducible from pCT [76].

Moreover, tumor perfusion parameters can show a decrease in advanced GC cases, suggesting that they play a role in the identification of more aggressive subtypes, as previously reported [44]. The authors compared three groups with different degrees of GC demonstrating significant differences in terms of blood flow (BF), blood volume (BV) and the permeability surface (PS) when comparing well-, intermediate, and poorly differentiated GC.

### 4.3. Radiomics and Artifical Intelligence (AI)

During previous decades, radiomics has been considered a widely applicable technique for different pathological conditions that may also play a role in the detection of GC. Firstly, radiomics can aid in the differential diagnoses between GC subtypes: a study of 171 patients demonstrated that radiomic signature had AUC values of 0.755, 0.710 and 0.712 in training, internal and external validation cohorts, respectively [77].

Moreover, a recently published systematic review [78] enrolled 25 studies including a total of more than 10000 patients. The most commonly used imaging technique was CT (96% of the included studies). The authors reported that radiomics is particularly useful for determining the treatment response. One of the most important published papers [79], which enrolled 292 patients, demonstrated that a CT-based radiomics model could predict the early detection of pathological downstaging following neoadjuvant chemotherapy in advanced GC.

Promising results were found also in a study of 955 patients [80] which aimed to predict peritoneal dissemination in GC: the authors demonstrated that the radiomic signature can be considered an independent predictor in both test and validation cohorts.

Similarly, AI plays a potential role in the evaluation of GC patients. AI techniques can be applied to the diagnosis of GC, especially by using EUS: different systems may play roles in increasing the accuracy of early detection of the primary tumor [81]. Moreover, deep learning can increase the accuracy of the detection of peritoneal dissemination. A recent multicenter retrospective study, enrolling 1978 patients, reported that the model achieved an AUC of 0.946, with good overall sensitivity and specificity levels (75.4% and 92.9%), higher than clinicopathological factors (AUC = 0.510–0.630) [82].

AI can be used to predict treatment outcomes, and more promising results were published in 2021 [83]. The authors, by enrolling 2209 patients, demonstrated that the deep-learning model had a high diagnostic accuracy in the assessment of tumor stroma (AUC = 0.960): this factor can be considered an independent predictor of DFS and OS in test and validation cohorts.

## 5. Future Directions

The application of different imaging techniques can help to stage gastric cancer patients, in particular, by using MRI, thanks to its high soft-tissue resolution and the usefulness of DWI. In this regard, further studies should be focused on the application of MRI in the detection and characterization of primary tumors and local nodes. Moreover, new radionuclides, including DOTA, should be deeply evaluated to better determine their applicability in clinical practice.

## 6. Conclusions

Although CT remains the preferred diagnostic technique for GC staging, along with EUS, which is the most accurate method for EGC, all of the diagnostic techniques described are fundamental for the comprehensive evaluation of patients with GC and to better determine methods for their appropriate management. Thus, the accuracy of CT and EUS could be certainly improved through the use of VG, MRI, volumetric CT and pCT, especially when distinguishing between nonadvanced and advanced GCs, for the evaluation of distant metastasis and for the assessment of treatment response.

## Figures and Tables

**Table 1:** Details of the most important papers regarding the usefulness of EUS in the staging of GC patients.

Ref #	Manuscript Type	Main Findings
[19]	Meta-analysis	- EUS results depend on the disease stage - Pooled sensitivity levels for T1 and T2 were 88.1 and 82.3%, respectively, while they were 89.7 and 99.2% for T3 and T4 - Pooled sensitivity levels for N1 and N2 were 58.2% and 64.9%
[20]	Original study	- The accuracy for detecting intramucosal cancer was 78% - Accuracy was lower in lesions located in the upper stomach
[21]	Original study	- Overall accuracy of 74.7% - Accuracy for T staging of 61.7% - Accuracy for N staging of 66%
[22]	Original study	- EUS and CT produce comparable results regarding T and N staging - EUS produces better staging of T2 and T3 lesions

**Table 2:** CT criteria for T staging in gastric cancer patients. Adapted from [24].

Stage	Pathological Features	CT Features
T1	lesion invades the lamina propria, muscularis mucosae and submucosa	- focal thickening in the inner and/or middle layer with strong enhancement - enhancement of the stomach walls without thickening - focal thickening with intense enhancement of the inner layer associated with a hypoattenuating peripheral layer
T2	lesion invades the muscularis propria	- thickening of the whole stomach walls with a regular outer surface - normal appearance of perigastric fat
T3	lesion invades the subserosa	- thickening of the whole stomach walls with a regular outer surface - normal appearance of perigastric fat
T4a	lesion invades the serosa	- thickening of the whole stomach walls with homogeneous or inhomogeneous enhancement - irregular outer surface - perigastric fat stranding - irregular nodules in the perigastric fat
T4b	lesion invades adjacent structures	- features of T4a stages - absence of fat planes between the primary tumor and adjacent organs or structures

**Table 3:** Details of the most important papers regarding the usefulness of CT in the staging of GC patients.

Ref #	Manuscript Type	Main Findings
[24]	Original study	- Overall sensitivity of 87% - Overall accuracy with axial images of 77% - Overall accuracy with VG of 84% - No differences between axial and 3DE images for N staging
[29]	Original study	- Detection rates with axial images, MPR, and in combination were 91%, 95%, and 98% - Assessment of tumor invasion was higher with MPR images - No differences were found regarding N staging
[31]	Original study	- Accuracy was high, especially for T1 (94.3%) and N2 (87.3%) - CT can be considered a preoperative predictor factor
[32]	Original study	- Accuracy of axial, MPR, and VG was 85.8%, 87.9%, and 92.8% - VG can help with GC detection, particularly in the case of EGC
[36]	Original study	- Accuracy for N staging was 86.3% - Acceptable reliability analysis between CT and pathological analysis
[37]	Original study	- Sensitivity and specificity levels for detecting peritoneal metastases were 28.3% and 98.9% - Laparoscopy remains the gold standard for peritoneal metastasis detection
[38]	Original study	- The amount of ascites identified on CT was an independent predictor factor of peritoneal metastases

**Table 4:** Details of the most important papers regarding the usefulness of MRI in the staging of GC patients.

Ref #	Manuscript Type	Main Findings
[44]	Meta-analysis	- MRI performed better in preoperative staging in comparison with CT - Agreement between pre- and postoperative TNM staging was not perfect
[45]	Meta-analysis	- Good diagnostic accuracy for preoperative T staging - Fair diagnostic accuracy for preoperative N staging
[46]	Original study	- The overall sensitivity levels of CT and MRI are comparable - MRI accuracy was higher for T1 and T2 (50% vs. 37.5% and 81.2% vs. 68.7%)
[48]	Meta-analysis	- MRI had a similar per-patient diagnostic accuracy to PET - MRI did not outperform CT in terms of staging
[49]	Original study	- DWI can increase the sensitivity of T and N staging - Preoperative ADC values correlated with pT staging - A significant difference in ADC between ≤T3 and T4 stages was found
[50]	Original study	- The sensitivity of DWI in N staging was 75%, 79.3%, and 60% for N1, N2 and N3 - The specificity of DWI in N staging was 84.6%, 77.3%, and 97.6%, for N1, N2, and N3
[51]	Original study	- The accuracy of MRI increased by adding DWI in comparison with using T2WI and contrast-enhanced sequences alone (88.2% vs. 76.5%)
